# Venous thoracic outlet syndrome secondary to arterial stent implantation: A case report

**DOI:** 10.1097/MD.0000000000017829

**Published:** 2019-11-22

**Authors:** Yang Liu, Zhoupeng Wu, Bin Huang, Yi Yang, Jichun Zhao, Yukui Ma

**Affiliations:** a Department of Vascular Surgery, West China Hospital; b West China School of Medicine, Sichuan University, Chengdu, China.

**Keywords:** arteriovenous malformation, endovascular, stent, thoracic outlet syndrome

## Abstract

**Rationale:**

Venous thoracic outlet syndrome (VTOS) secondary to subclavian arterial stent implantation is extremely rare. Here, we firstly report this disease and the endovascular intervention using covered-stents.

**Patient concerns:**

An 80-year-old man who had received an acceptable stent implantation for the treatment of a right subclavian arteriovenous malformation (AVM), presented with a gradually increasing swelling and pain in his right upper extremity.

**Diagnosis:**

The patient was diagnosed with right VTOS and recurrent subclavian AVM following ultrasonography and computed tomographic angiography.

**Interventions:**

We positioned a covered-stent in the subclavian artery to block the feeding arteries and successfully embolized the remaining branches with coils. Next, we performed successful dilation 3 times, followed by the positioning of another covered-stent in the right subclavian vein.

**Outcomes:**

The patient was free of all symptoms and the imaging procedures confirmed an acceptable thrombosis of the AVM with patent stents in the right subclavian artery and vein during the 6-month follow-up.

**Lessons:**

Venous stent implantation is an alternative to treat VTOS caused by subclavian arterial stents and it is essential to pay more attention to the incidence of VTOS following arterial stent implantation in the subclavian artery.

## 1 Introduction

Venous thoracic outlet syndrome (VTOS), a type of thoracic outlet syndrome, is described as a constellation of complex symptoms resulting from extrinsic subclavian vein compression at the upper thoracic aperture. VTOS is relatively rare, with an annual incidence of about 10/100,000 and accounts for 4% to 5% of all thoracic outlet syndromes.^[1]^ The major symptoms include arm pain, swelling, heaviness, discoloration, and the development of venous collaterals in the upper extremities. The presence of structural anomalies such as an abnormal first rib, cervical rib, clavicular deformity, hypertrophied anterior scalene musculature and tendons, are usually responsible for the extrinsic compression at the costoclavicular junction or the pectoralis minor space.^[2]^ The extrinsic compression could also be a result of individual habits such as repetitive movements, strenuous exercise, and positional maneuvers during athletics or some occupations. In addition, a soft tissue mass around the subclavian vein is another rare cause.^[3]^ However, VTOS as a result of compression by an implanted stent in the subclavian artery has not been previously reported in the literature. Here, we report a chronic VTOS secondary to arterial stent implantation for subclavian arteriovenous malformation (AVM) and discuss the proper treatment for an 80-year-old male patient. We have obtained written informed consent from the patient and his family for the publication of this case report.

## 2 Case report

**Figure 1:**
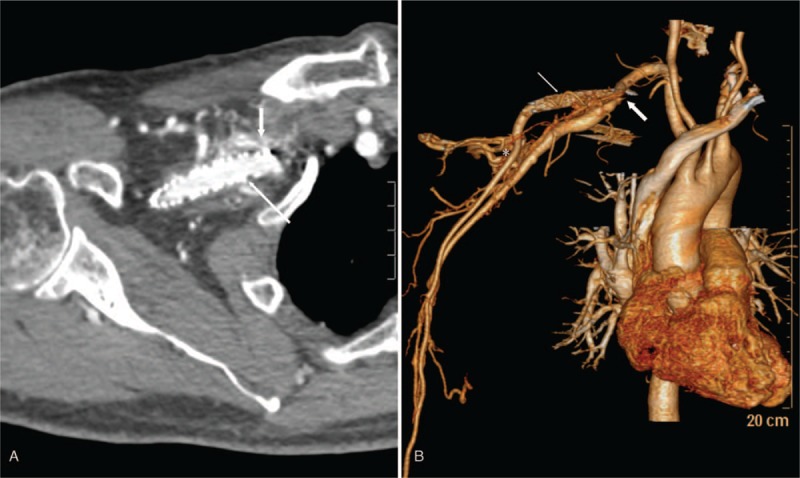
Computed tomographic angiography showed the stent in the subclavian artery (thin arrows in A and B), the occluded subclavian vein compressed by the stent (thick arrows in A and B), and the multiple arteriovenous malformations between the right subclavian artery and vein (asterisk).

An 80-year-old man who had received an acceptable stent implantation (10 mm × 60 mm Fluency stent, Bard Incorporation, Germany) 2 years ago for the treatment of a right subclavian AVM, was admitted to our hospital with complaints of a gradually increasing swelling and pain in his right upper extremity over the past 3 months. The patient denied a history of trauma, infection, and neoplasm. Physical examination showed that his right upper extremity displayed extensive swelling, hyperpigmentation, and subcutaneous palpable varicosities. The motor and sensory functions were normal. Ultrasonography and computed tomographic angiography (CTA) showed that the right subclavian vein was occluded due to compression of the stent in the subclavian artery and there were multiple arteriovenous communications between the right subclavian artery and the adjacent veins (Fig. 1A and B). A diagnosis of VTOS with recurrent AVM was suspected.

**Figure 2:**
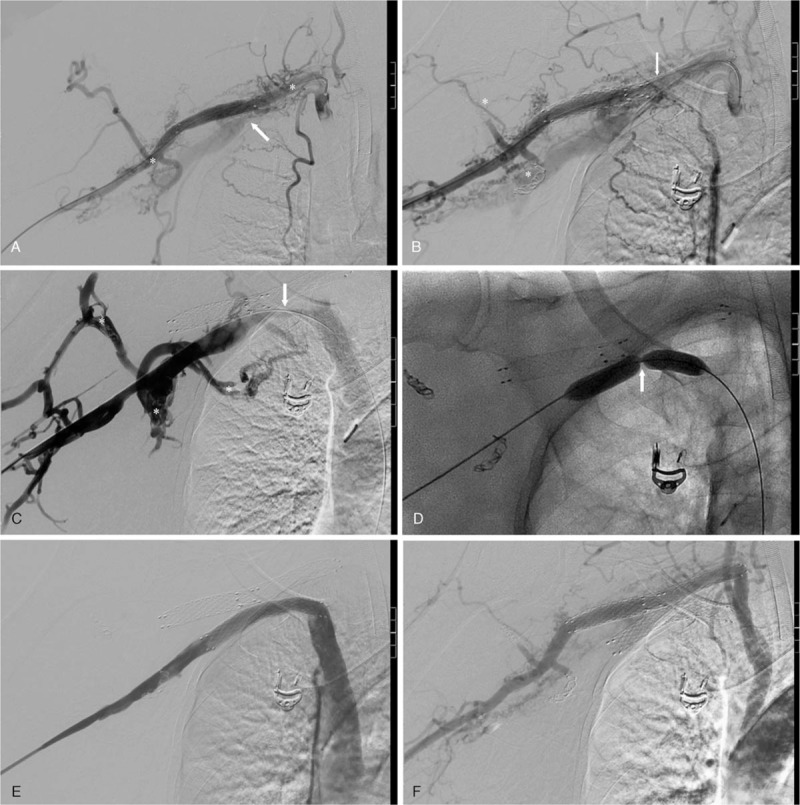
The details of endovascular procedure. A. Arterial angiogram showed the multiple arteriovenous malformations (asterisks), and the abnormal subclavian vein (arrow); B. We successfully positioned a covered-stent in the subclavian artery (arrow) and embolized the remaining branches with coils (asterisks); C. Venous angiogram showed the multiple arteriovenous malformations (asterisks), and the occluded subclavian (arrow); D. The sharp incisura emerged when the balloon was inflated in the subclavian vein (arrow); E. Venous confirmatory angiogram demonstrated a patent subclavian vein after the position of covered-stent; F. Arterial confirmatory angiogram demonstrated that the subclavian artery was patent, the subclavian vein did not emerge in advance, and most of arteriovenous nidus had completely disappeared.

The patient was scheduled for endovascular therapy following extensive deliberation. An angiogram of the artery showed that multiple feeding arteries arising from the right subclavian artery formed connections with the adjacent veins, and the subclavian vein emerged in advance (Fig. 2A). We positioned a 10 mm × 80 mm fluency stent in the subclavian artery to block the feeding arteries and successfully embolized the remaining branches with coils (Fig. 2B). Next, an angiogram of the vein demonstrated complete occlusion of the right subclavian vein near the previous arterial stent and a mass of enlarged veins (Fig. 2C). During the procedure, when the balloon was inflated in the subclavian vein, a sharp incisura emerged from the narrowest vein segment (Fig. 2D). We performed successful dilation three times (lasting 2 minutes each time), followed by the positioning of another 10 mm × 60 mm Fluency stent in the right subclavian vein. The last confirmatory angiogram demonstrated that both the stents as well as the subclavian vessels, were patent and in normal condition, and most of AVM nidus had completely disappeared (Fig. 2E and F).

**Figure 3:**
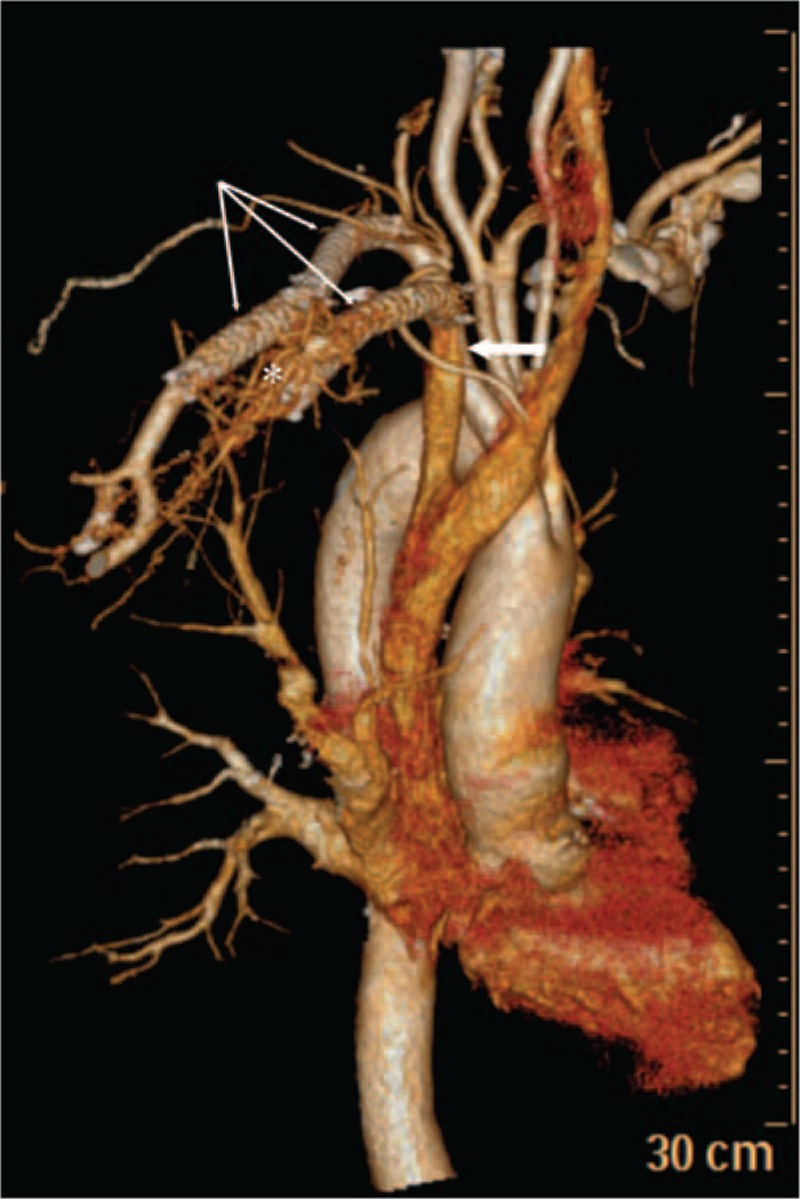
Computed tomographic angiography during 3-month follow-up showed the patent stents in the right subclavian artery and vein (thin arrows), the patent subclavian vein (thick arrow), and an acceptable thrombosis of the multiple arteriovenous malformations (asterisk).

**Figure 4:**
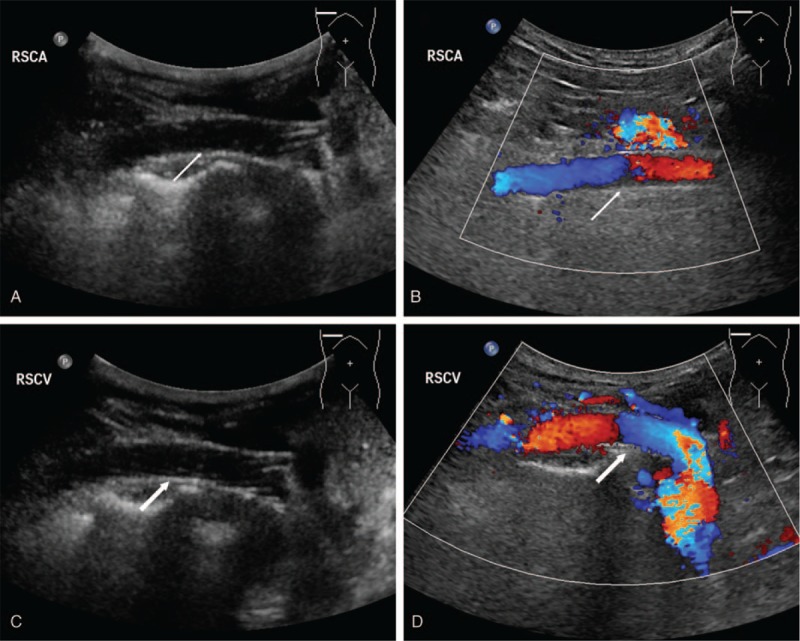
Ultrasonography image during 6-month follow-up showed the patent stent in the right subclavian artery (thin arrows in A and B) and the patent stent in the right subclavian vein (thick arrows in C and D).

The patient was provided anticoagulation therapy after the operation, and discharged 3 days later, after significant relief from the symptoms. During the 6-month follow-up, the patient was free of all symptoms and the imaging procedures confirmed an acceptable thrombosis of the AVM with patent stents in the right subclavian artery and vein (Figs. 3 and 4).

## 3 Discussion

VTOS is caused by extrinsic compression of the subclavian vein in the thoracic outlet area, usually resulting from anatomic abnormalities, repetitive shoulder movements, and the presence of soft masses. However, VTOS secondary to a subclavian arterial stent implantation is extremely rare, and to our knowledge, this is the first report.

In previous VTOS cases, the treatment was based on the severity of symptoms. Conservative management including anticoagulation, pain control, and arm compression can be provided for mild symptoms. However, this is usually associated with poor results such as thrombus recurrence and aggravating stenosis.^[4]^ For severe symptoms, the interventional treatment algorithm introduced by Kunkel and Machleder in 1989, is generally accepted in most hospitals.^[5]^ This stratified management includes catheter-directed thrombolysis, surgical first rib resection and scalenectomy, follow-up venography, and endovascular management.^[4,6–8]^

There are no existing studies that report venous stent implantation alone for VTOS, and in several cases the venous stents used were placed during the follow-up endovascular therapy after thoracic outlet decompression^[2,9,10]^ Considering that the main cause for venous stenosis is the direct compression from nearby abnormal structures, venous stent implantation without performing thoracic outlet decompression may easily lead to subsequent problems including stent fracture, migration, deformation, and thrombosis.^[2]^ In addition, according to previous reports, most of the VTOS patients are young and stent implantation may lead to challenges due to the relatively short life of the stents.^[4,6–8]^

However, in this case, a venous stent implantation was feasible. Before the procedure, a comprehensive protocol encompassing AVM resection, stent explantation, and vessels reconstruction was discussed considerably. Apart from compression by the subclavian arterial stent, the CTA showed no evidence of extrinsic compression from the first rib, clavicular deformity, hypertrophied anterior scalene musculature and tendons. Therefore, there was no need to perform the traditional treatment procedures for VTOS like first rib resection and scalenectomy. Considering the difficult eradicative resection and the uncontrolled blood loss from open surgery, we decided to treat this aged patient with a minimally invasive endovascular therapy for the AVM as well as VTOS. Since the venous occlusion was very tenacious, during the procedure a wire was inserted by intense repetitive attempts, and a sharp incisura emerged from the narrowest vessel segment after the balloon was inflated, which meant it could not be treated by catheter-directed thrombolysis alone. Since the extrinsic compression was caused by the arterial stent compression and not by the strong force exerted by an anomalous structure like a rib or clavicle, it indicated that the venous stent had sufficient mechanical force. In addition, unlike in younger patients, due to the advanced age of the patient, there were fewer complications anticipated from the stent service life. Therefore, we decided to choose a covered-stent not only to maintain the patency but also to block aberrant branches of the subclavian vein. More importantly, both the last confirmatory angiogram and the 6-month follow-up examination, demonstrated an excellent outcome. However, it is essential to emphasize the limitations regarding the unclear long-term outcomes of stent patency and AVM recurrence.

In summary, venous stent implantation is an alternative to treat VTOS caused by subclavian arterial stents and it is essential to pay more attention to the incidence of VTOS following arterial stent implantation in the subclavian artery.

## Author contributions

**Conceptualization:** Yang Liu, Bin Huang, Yukui Ma.

**Data curation:** Yang Liu, Yi Yang.

**Investigation:** Yang Liu, Zhoupeng Wu, Bin Huang, Yi Yang, Jichun Zhao, Yukui Ma.

**Methodology:** Yang Liu, Zhoupeng Wu, Bin Huang, Jichun Zhao, Yukui Ma.

**Project administration:** Yang Liu, Bin Huang, Yi Yang.

**Resources:** Bin Huang, Jichun Zhao, Yukui Ma.

**Software:** Zhoupeng Wu.

**Validation:** Yang Liu, Zhoupeng Wu, Bin Huang, Yi Yang, Jichun Zhao, Yukui Ma.

**Visualization:** Yang Liu, Zhoupeng Wu, Jichun Zhao, Yukui Ma.

**Writing – original draft:** Yang Liu, Zhoupeng Wu, Yi Yang.

**Writing – review & editing:** Bin Huang, Yukui Ma.
